# Sulfuryl Fluoride Fumigation as a Quarantine Treatment for the Control of *Reticulitermes speratus* Kolbe (Blattodea: Rhinotermitidae) in Wood

**DOI:** 10.3390/insects15060379

**Published:** 2024-05-22

**Authors:** So-Yeon Kim, Na-Ra Choi, Min-Goo Park

**Affiliations:** Department of Bioenvironmental Chemistry, Jeonbuk National University, Jeonju 54896, Republic of Korea; ksy000322@naver.com (S.-Y.K.); bade1030@naver.com (N.-R.C.)

**Keywords:** termites, methyl bromide alternative, phytosanitary treatment, efficacy, sorption and penetration

## Abstract

**Simple Summary:**

Sulfuryl fluoride (SF) was introduced as a fumigant in 1957 and widely used against stored product insects and dry wood termites in wood structures in warm climates. We conducted several experiments on the quarantine treatment of Japanese termites (*Reticulitermes speratus*) with SF. The efficacy against *R. speratus* in wood, as well as the wood sorption and penetration capacity, of SF were investigated. The LCt_50_ and LCt_99_ values of SF were calculated at two different temperatures and the differences between dry and wet wood at several loading ratios were evaluated as insignificant. Complete pest mortality was proven in scale-up trials with a 90% loading ratio of SF. The fumigant concentration decreased below the threshold limit after 30 min of ventilation. Compared with other alternative methyl bromide fumigants, the main advantages of SF were outlined. SF caused 100% termite mortality with a 90% loading ratio in the scale-up trials (500 L). The present study provides a basis for the use of SF as an alternative to MB for the treatment of termites in wood.

**Abstract:**

High-risk termites in wood imported to the Republic of Korea are currently treated with methyl bromide (MB), which has ozone-depleting properties and is highly toxic. This study evaluated the effectiveness of sulfuryl fluoride (SF) as a quarantine treatment against *Reticulitermes speratus* Kolbe (Blattodea: Rhinotermitidae) in wood, along with its wood sorption and penetration capacity. The LCt_50_ and LCt_99_ values for SF were 30.87 and 42.53 mg h/L at 23 °C and 151.62 and 401.9 mg h/L at 5 °C, respectively. The SF Ct values did not significantly differ between dry and wet wood at loading ratios of 10%, 30%, and 50% at both 5 °C and 23 °C (*p* > 0.05). In a closed wooden cube, the LCt_50_ and LCt_99_ for SF for *R. speratus* were 31.59 and 53.34 mg h/L, respectively, indicating an excellent wood penetration ability. SF caused 100% termite mortality with a 90% loading ratio in the scale-up trials (500 L). The SF concentration during ventilation decreased below the threshold limit value (TLV) of 5 ppm within 30 min, confirming that the working conditions were safe. This study provides a basis for the use of SF as an alternative to MB for the treatment of termites in wood.

## 1. Introduction

Termites are important pests that affect wood [1]. Some species consume wood, causing severe damage to wooden structures. They are often only detected when noticeable changes appear on the wood surface, by which time extensive damage has occurred to the interior of the wood [2]. Recently, *Incisitermes minor* Hagen (Blattodea: Kalotermitidae) and *Cryptotermes domesticus* Haviland (Blattodea: Kalotermitidae) have invaded urban areas in the Republic of Korea (Korea) from abroad. This is a critical issue because of their ecological characteristics, which enable them to survive in low-moisture environments [3,4]. *Reticulitermes speratus* Kolbe (Blattodea: Rhinotermitidae), one of the termites distributed within Korea [5], has inflicted significant harm to wooden structures, including culturally significant sites like temples and palaces [6]. Globally, termite infestations result in economic losses exceeding USD 20 billion annually [7].

When high-risk termites are found in wood imported to Korea, methyl bromide (MB) fumigation is used for disinfection according to current regulations [8]. Approximately 300 tons of MB was used annually for wood quarantine treatment from 2019 to 2021, which was almost 75% of the total amount of MB consumed in Korea [9]. MB was classified as an ozone-depleting substance under the Montreal Protocol, which was established in 1989 [10], and the International Plant Protection Organization (IPPC) recommended its replacement and reduction [11]. Furthermore, MB is a highly toxic pesticide, and recent studies have reported that, even without obvious symptoms, workers experience negative effects in relation to their central nervous, autonomic nervous, and vascular systems [12,13,14]. Korea has developed MB alternatives such as phosphine (PH_3_) and ethyl formate (EF), which have been used in quarantine procedures since 2011, and ethanedinitrile (EDN), which was introduced in 2019 [15,16,17]. However, these alternatives have some disadvantages. EF has a high sorption rate and lower permeability because of its lower vapor pressure compared with other fumigants [18]. PH_3_ requires a longer fumigation time than MB or EDN [16]. EDN is expensive, costing more than twice that of MB, without a significant difference in the treatment efficacy or convenience of use [19]. Consequently, the use of MB has not decreased, despite the availability of alternatives.

SF effectively controls various wood pests, including wood borers, bark beetles, and termites, and has been used globally [20,21,22]. However, SF is not currently approved as a substitute for MB in Korea [8]. For quarantine treatment, the Probit 9 efficacy— a 99% mortality rate or higher through treatment—should be confirmed by the Animal and Plant Quarantine Agency (APQA) in Korea [8], ensuring compliance with the IPPC [23]. Demonstrating the efficacy of SF for termite control requires several steps [23].

To address this, our study assessed the feasibility of utilizing SF instead of MB in controlling termites in wood. *R. speratus,* a termite species previously untested against SF, was selected as a representative invasive termite in this study. In this study, the efficacy of SF on *R. speratus* was evaluated in small-scale laboratory experiments (desiccators < 14 L) at 5 °C and 23 °C. The SF sorption was analyzed in both dry and wet wood across different loading ratios (10%, 30%, and 50%) and temperatures (5 °C and 23 °C) to determine the optimal loading ratio for scale-up trials. The SF penetration was assessed by evaluating the mortality of *R. speratus* in wooden cubes. Finally, scale-up trials were conducted using a 0.5 m^3^ (500 L) fumigation chamber with *R. speratus* specimens.

## 2. Materials and Methods

### 2.1. Fumigant, Insect, and Wood

Sulfuryl fluoride (99% purity, analytical grade) was supplied by KGLOBAL Co. (Iksan, Republic of Korea) and stored in a gas tank.

Workers of *R. speratus* were collected from a wild population, which had not been treated with any insecticide, in Yeoju and Jinju, Korea, and then stored at the Jeonbuk National University in a rearing room maintained at 25 ± 1 °C with 60–70% relative humidity (RH) and 16:8 (L:D). We conducted tests within 6 months of collection. The termites were identified by a specialist from the APQA in Korea. Pine and oak wood pieces were provided as food.

Wood was purchased as timber imported to Korea from abroad by UniB & C (Seoul, Republic of Korea) for the sorption and scale-up tests.

### 2.2. Sulfuryl Fluoride Concentration and Determination of Ct (Concentration × Time) Product

The SF concentration during fumigation was measured using a QP 2010 Plus GC/MS (Shimadzu, Tokyo, Japan) after separation on a GC-GASPRO column. The temperatures of the oven, injector, and detector were set at 90 °C, 100 °C, and 250 °C, respectively. The carrier gas was He. The SF concentration was measured by comparing the peak area to external SF gas standards. The concentrations were checked at intervals of 0.1, 0.5, 1.0, 2.0, 4.0, and 24 h of exposure in the fumigation chamber. The Ct products were calculated as described by Ren et al. [24], as follows:

(1) $$Ct=\sum \frac{\left(C_{i}+C_{i+1}\right)\left(t_{i+1}-t_{i}\right)}{2}$$

where C is the fumigant concentration (mg/L), t is the exposure time (h), i is the order of measurement, and Ct is the concentration × time product (mg h/L).

### 2.3. Efficacy of SF for the Fumigation of Reticulitermes speratus in Laboratory Trials

The SF fumigation of *R. speratus* was conducted in 6.9 L glass desiccators (Duran^®^, DWK life science, Millville, NJ, USA) or 14 L acrylic desiccators (UniB & C, Seoul, Korea) equipped with a small fan at the base for air circulation. We used two types of desiccators, selecting the size based on the fumigant dose: small desiccators were used for low doses and large desiccators for high doses. Vacuum grease was used to seal the glass desiccators. Termites were placed on Petri dishes (diameter 55 mm) covered with a mesh screen with a pore size of 0.053 mm. Wet filter papers were placed in the Petri dishes to provide food and moisture, and the Petri dishes were positioned in the desiccators. SF was injected into the desiccators using a syringe (SGE Analytical Science, Victoria, Australia) with scheduled doses of 1.2, 1.8, 2.0, 2.1, 2.2, 2.3, 2.4, 2.5, and 5.0 mg/L. The fan was operated for 1 h to assist with SF circulation. Gas sampling inside the desiccator was conducted for 24 h. After fumigation, the desiccators were opened and allowed to aerate for one hour in a fume hood. Treated termites were transferred to a rearing room under conditions of 25 ± 2 °C and 60–70% RH. Mortality was observed 24 h after fumigation. A procedure similar to the trial conducted at 23 °C was conducted at 5 °C, with SF dosages of 2, 5, 10, 15, 18, and 20 mg/L. Twenty workers were used in each replication. All treatment and control groups were replicated three times.

### 2.4. Sulfuryl Fluoride Sorption with Different Loading Ratios of Dry and Wet Wood

The SF sorption experiments for wood were conducted using pine wood blocks (5 × 6 × 6 cm) obtained from UniB & C Co. Ltd. The dry wood was used as purchased, whereas the wet wood was submerged in water for two weeks before use. The moisture content was determined by measuring the initial weight of each wood block before heating in the oven. After fumigation, the wood blocks were heated in a dry oven at 103 °C for 7 h and their masses were measured. This process was repeated five times, and the moisture content of both types of wood was measured. The moisture content calculation for the two types of wood was as follows [13]:

(2) $$Moisture\left(\%\right)=\frac{weight\;of\;evaporated\;moisture}{sample\;weight}\times 100(\%)$$

Both types of wood were placed in desiccators with reference to the loading ratios (10%, 30%, and 50% *v*/*v*). For the SF sorption test, a dosage of 30 mg/L of SF was applied. This was based on the LCt_99_ value obtained from a 5 °C efficacy test for *R. speratus*, as high doses facilitated sorption evaluation. After SF was injected into the desiccators, gas samples were collected at regular intervals (0.1, 0.5, 1.0, 2.0, 4.0, 6.0, and 24.0 h). All treatments were replicated three times, and the concentration of SF was measured using GC/MS.

### 2.5. Penetration Tests Based on R. speratus Mortality in Wooden Cubes

To assess the gas penetration capability, a pine wooden cube (10.0 × 10.0 × 10.0 cm) was prepared and modified following the techniques described by Park et al. [25]. The wooden cube consisted of two halves (bottom and top; 10 × 10 × 5 cm). Both halves of the cube were cut across the grain, and square hollows (2.0 × 2.0 × 1.0 cm) were created on one side of the central cube, perpendicular to the grain, either by drilling or chiseling. Thus, an insect chamber (2.0 × 2.0 × 2.0 cm) was created at the center of the two cubes when their hollow parts were joined together face to face for the fumigant penetration and bioassay investigation. Then, 10 to 20 workers of *R. speratus* were placed in the chamber inside one wooden cube. The two halves of the wooden cubes were joined together with a rubber gasket in between and secured with a snap fastener. Each of these sealed cubes, housing *R. speratus*, was then placed separately into 6.8 L fumigation desiccators. The SF dose range was from 0.5 to 3.5 mg/L at 23 °C for a 24 h holding period.

### 2.6. Scale-Up (0.5 m^3^) Fumigation Using SF on R. speratus

**Figure 1:**
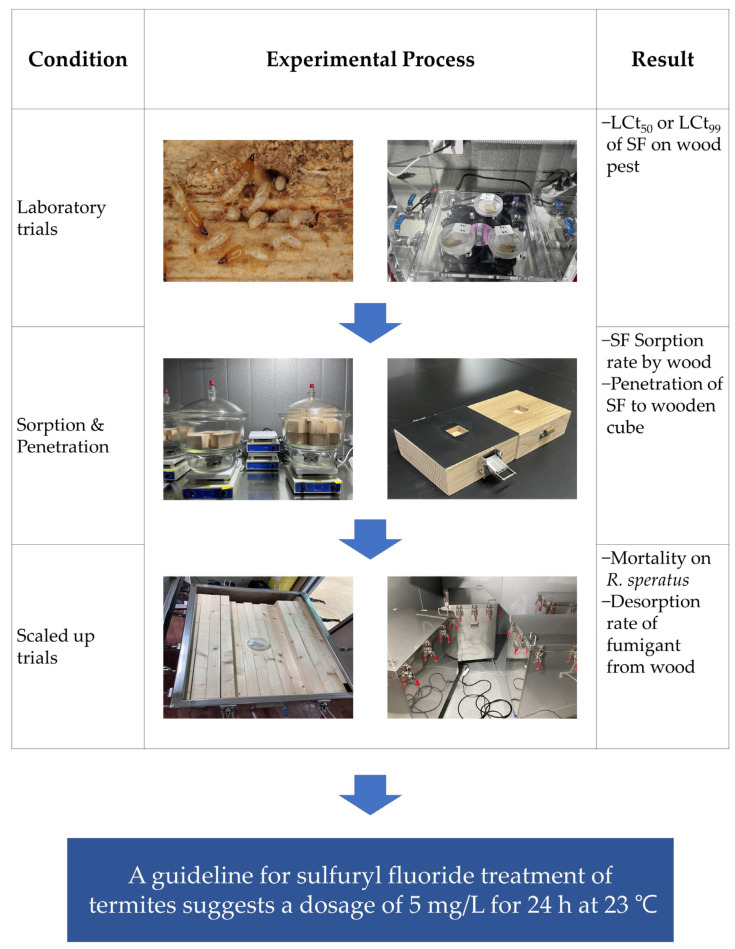
Illustrated methodology for development of phytosanitary fumigation schedule and suggested phytosanitary treatment guidelines.

Scale-up trials were conducted in a 0.5 m^3^ (500 L) stainless fumigation chamber (100 cm × 62.5 cm × 80 cm) at the Iksan, Jeonbuk Province. A total of 420 pine blocks (3 cm × 4 cm× 90 cm) were arranged in the chamber to achieve a loading ratio of 90% (calculated as 450 L/500 L × 100), which was the maximum loading ratio for the chamber to simulate commercial container fumigation with a high loading ratio. The termites in the Petri dishes were placed in the stainless chambers [20]. The chambers were kept in a temperature-controlled room (18 m^3^). SF was injected at a rate of 5 mg/L based on the efficacy test data. After SF fumigation at 23 °C for 24 h, the fumigation chambers were aerated for 1 h. The gas concentration in the room was monitored for 0.1, 0.5, and 1.0 h post-fumigation to evaluate the SF desorption in the air. The same GC/MS instrument described in Section 2.2 was used to analyze the concentrations. Air samples were collected using 1 L gas bags (SKC Tedlar bag, Eighty Four, PA, USA) at predetermined intervals within the room. After the fumigation, the treated termites were moved to a rearing room maintained at 25 ± 1 °C and 60–70% relative humidity to monitor their mortality. Each replication used 100 termites. All treatment and control groups in each experiment were replicated three times. Adjusted mortality rates were calculated using Abbott’s formula [(mort. treatment − mort. of control)/(1 − mort. of control) × 100]. The methodology used to develop the phytosanitary fumigation guidelines is illustrated in Figure 1.

### 2.7. Statistical Analysis

The dose–response relations of *R. speratus* and SF were analyzed using Probit analysis (Finney, 1971) [26], with a computer program developed by Ge Le Pattourel (Imperial College, London) and utilized by Don-Pedro (1989) [27]. In the Probit parameters, ‘slope’ signifies the correlation between the Ct products and the lethality of the termites; ‘*df*’ denotes the degrees of freedom related to the number of treated cases. LCt_50_ and LCt_99_ represent the Ct products where 50% and 99% lethality of the termites are attained, respectively. The wood sorption of the SF concentration in relation to the loading ratio and temperature was analyzed using a two-way ANOVA and t-test (SPSS ver. 23).

## 3. Results

### 3.1. Efficacy of SF on Reticulitermes speratus in Laboratory Trials

The efficacy of SF on *R. speratus* workers is shown in Table 1. The LCt_50_ and LCt_99_ values of SF were 30.87 and 42.53 mg h/L at 23 °C and 151.62 and 401.9 mg h/L at 5 °C, respectively.

### 3.2. Sulfuryl Fluoride Sorption with Various Loading Ratios for Dry and Wet Wood

The moisture content of the dry and wet wood was 12.41 ± 0.01% and 70.18 ± 0.12%, respectively. The SF Ct values did not significantly differ between dry and wet wood at loading ratios of 10%, 30%, and 50% at both 5 °C and 23 °C (*p* > 0.05 for all cases). This indicates that the moisture content of the wood did not affect the SF sorption. Furthermore, the Ct products were not significantly different with the loading ratios of 10%, 30%, and 50% at 5 °C and 23 °C (*p* > 0.05 except for wet conditions at 5 °C), which indicates that the loading ratio did not affect the SF sorption. Details are shown in Table 2.

### 3.3. Penetration Test Based on Reticulitermes speratus Mortality in a Wooden Cube

Within the closed wooden cube, the LCt_50_ and LCt_99_ of SF on *R. speratus* were 31.59 mg h/L and 53.34 mg h/L, respectively (Table 3). Without the wooden cube, the LCt_50_ and LCt_99_ values were 30.87 mg h/L with a 95% CI of 29.65–32.02 and 42.53 mg h/L with a 95% CI of 39.714–7.49 (Table 1). The LCt_50_ and LCt_99_ values were not significantly different in both conditions, indicating that SF has an excellent wood penetration ability.

### 3.4. Scaled-Up Fumigation Using Sulfuryl Fluoride on Reticulitermes speratus

**Figure 2:**
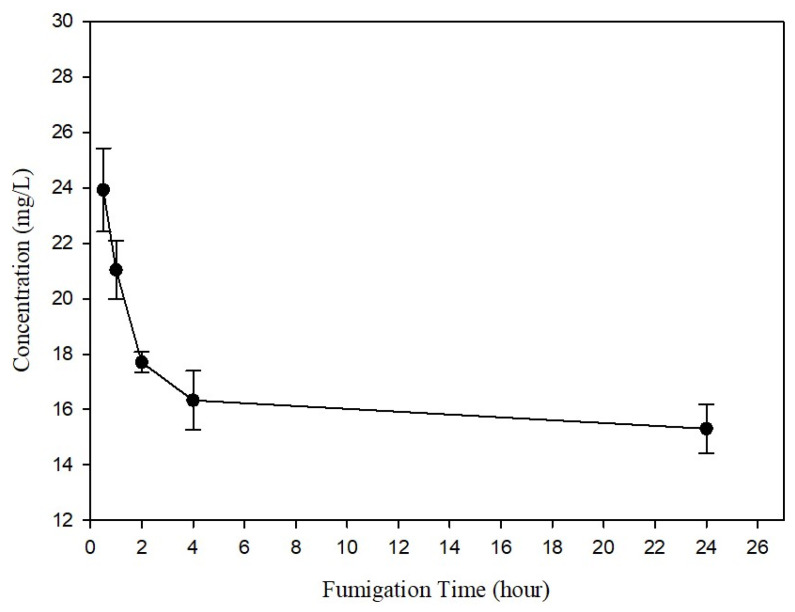
Sulfuryl fluoride concentration in fumigation chambers (0.5 m^3^) for 24 h during scale-up trials.

**Figure 3:**
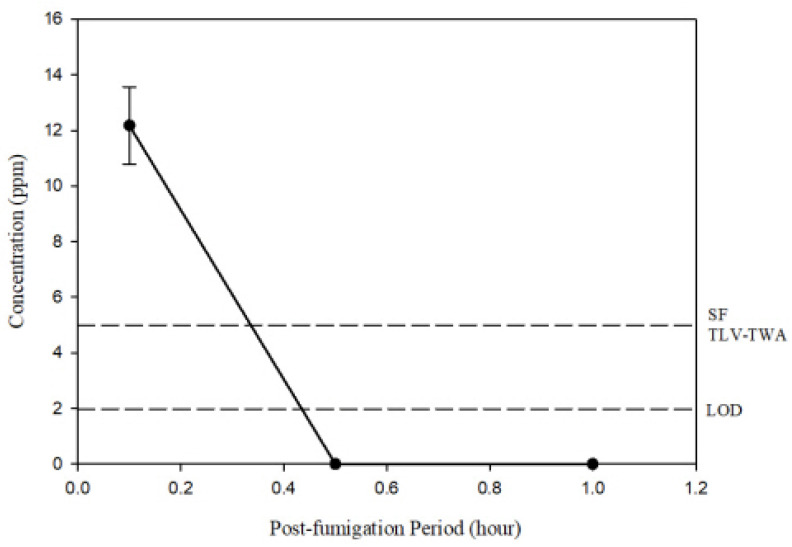
Sulfuryl fluoride concentration in 18 m^3^ room for 1 h following conclusion of fumigation in scaled-up trials. The concentration decreased to below the threshold limit value (TLV-TWA) of 5 ppm within 0.5 h.

Based on the efficacy trials and sorption test with *R. speratus*, workers were fumigated at a concentration of 5 mg/L at 23 °C with a 90% loading ratio in a stainless chamber (0.5 m^3^) for 24 h. SF caused 100% termite mortality and no mortality occurred in the control. The concentrations 0.5 h and 24 h after the SF injections were 29.13 ± 7.9 mg/L and 15.31 ± 1.53 mg/L, respectively, which are greater than the injected dose of 5 mg/L (Figure 2). The LCt_99_ was 419.1 ± 40.41 mg h/L, indicating values of more than 42.53 mg h/L of the LCt_99_ on the termites at 23 °C and 401.9 mg h/L at 5 °C in the laboratory trials. After the 24 h fumigation, the SF concentration inside the room, which included the fumigation chambers, decreased from 12.18 ± 2.4 ppm to below the limit of detection (1.98 ppm) within 0.5 h of ventilation (Figure 3). This reduction below the exposure limit (e.g., the threshold limit value for time-weighted average on the basis of an 8 h work day and a 40 h work week, TLV-TWA) of the SF threshold limit value (TLV) of 5 ppm confirmed the safe conditions for re-entry for work [28,29].

## 4. Discussion

This study shows that SF is as effective as MB in controlling termite infestations in imported timber. SF was also found to have excellent permeability and low sorption. The concentration for the control of *R. speratus* was 42.53 mg h/L at 23 °C and 401.9 mg h/L at 5 °C. The complete disinfection of the termites was confirmed using scaled-up conditions in a 0.5 m^3^ stainless chamber with 5 g/L at 23 °C. After the wood was fumigated for 24 h, the SF concentration decreased to < 5 ppm (TLV-TWA of SF) after 0.5 h of ventilation, indicating a high level of worker safety.

According to previous SF research in relation to other termite species, injections of approximately 2–2.5 mg/L (Ct 44–55 mg h/L) at 27 °C resulted in a 99% mortality rate for *Coptotermes formosanus* Shiraki (Blattodea: Rhinotermitidae)*, Incisitermes snyderi* Light (Blattodea: Kalotermitidae), and *I. minor* [30]. *C. cavifrons* and *I. schwarzi* were successfully killed at 30–46 mg h/L at 27 °C [20]. The LCt_99_ of SF on *R. speratus* in this study was 42.53 mg h/L at 23 °C, aligning with the results from previous studies. This confirms that the LCt_99_ is effective in disinfecting wood affected by various termites. Furthermore, this indicates that higher doses of SF are required to control termites at lower temperatures as the LCt_99_ of SF was 401.9 mg h/L at 5 °C.

As an MB alternative on wood, EDN’s Ct products were found to be 249.7 g h/m^3^ with moisture content of 54.5% and 175.7 g h/m^3^ with moisture content of 21.1% at 22 °C and a loading ratio of 20% [25]. To increase the loading ratio from 20% to 40%, the Ct products from the EDN were decreased from 249.7 g h/m^3^ to 146.5 g h/m^3^ at 22 °C [25]. The lower moisture content and higher loading ratio resulted in increased EDN absorbance. This means that the efficacy of the treatment varies depending on the moisture content and loading ratios, but SF was not affected by the loading ratio or moisture content (Table 2). The Ct products showed no significant differences between dry and wet wood, or among the loading ratios of 10%, 30%, and 50% (*p* > 0.05 for all cases). PH_3_ exhibited a similar trend to SF in sorption between dry and wet wood [16]. Thus, SF is more applicable for field trials with various moisture conditions in the wood or a high loading ratio, because sorption is not a problem for SF, unlike other fumigants.

The penetration of fumigants is crucial for thorough pest control and varies depending on the type of product used [31,32]. In previous studies investigating the penetrative properties of different treatments on timber, MB showed limited penetration, as, even after 24 h of exposure, the concentration within the timber blocks did not significantly increase compared with the chamber’s concentration [24]. In addition, the LCt_99_ of MB for the control termites in a wooden box and in the open state was 112.91 mg h/L and 86.92 mg h/L, respectively, indicating low permeability [25]. In contrast, SF penetrated timber blocks more effectively than MB, demonstrating its superior penetration [24]. Similarly, the efficacy test of SF in this study showed an LCt_99_ value of 42.53 mg h/L in open conditions and 53.34 mg h/L in wooden cube conditions at 23 °C, showing the high penetrability of the SF due to the lack of significant differences in the Ct values.

The residues left after fumigation can impact worker safety and the surrounding environment. In a previous study on orange fumigation, the internal concentrations in oranges stored after fumigation were maintained at 12.6 to 36.6 ppm and 9.4 to 32.5 ppm for EF and MB, respectively [33]. While the EF concentrations remained below the TLV of 100 ppm, MB exceeded its TLV of 1 ppm [33]. Similarly, in sweet persimmon fumigation research, the EF concentration in unpackaged fruit decreased to <100 ppm (EF’s TLV) within 1 h [34]. In this study, the concentrations outside the chamber were found to be lower than the TLV of SF (5 ppm) within 1 h [29], with similarity to EF in the previous studies, which confirms that SF does not have a significant impact on worker safety. Experiences with structural fumigation for termite extermination in the USA have shown that low gas concentrations of 1 ppm can cause acute human reactions [35]. However, as exposure at 1 ppm a month following fumigation at home was more dangerous than that from the TLV-TWA calculated on the basis of an 8 h work day and a 40 h work week, and the level of SF in this study was almost zero at 30 min after the fumigation of the wood in the chamber, the fumigation conditions can be considered safe.

The restricted stage of the termites and the small-scale trials used to determine the efficacy of SF were both limitations of this study. Future research should evaluate the efficacy of SF with a greater number of pests in a large-scale trial to assess its application in actual quarantine conditions. Since this study only evaluated *R. speratus* workers, it is necessary to determine the SF concentrations for other stages, such as eggs.

Nevertheless, this study determined the appropriate concentration and duration of SF needed to efficiently manage the newly identified pest, *R. speratus*, in wood. The relevance of the findings from this study, including the sorption and penetration of SF on wood depending on the loading ratio and moisture content of the wood, is also highlighted. This research will serve as a basis for the development of phytosanitary treatments using sulfuryl fluoride, which will reduce the use of MB in the wood trade. In addition, the commercial application of environmentally friendly and worker-friendly fumigants in the wood trade will create new markets for MB alternatives and improve the safety of quarantine sites.

## Figures and Tables

**Table 1:** Efficacy of fumigation with sulfuryl fluoride on *Reticulitermes speratus* at 5 °C and 23 °C for 24 h in laboratory trials (6.9 L or 14.0 L).

Temp (°C)	No. of Termites	LCt_50_^a^ (95% CI, mg h/L)	LCt_99_^a^ (95% CI, mg h/L)	Slope ± SE ^b^	*df* ^c^	*X* ^2 d^
23	418	30.87 (29.65–32.02)	42.53 (39.71–47.49)	16.7 ± 2.1	25	63.61
5	480	151.62 (140.78–162.90)	401.9 (343.69–499.79)	5.5 ± 0.5	25	120.8

^a^ The LCt_50_ and LCt_99_ values, along with the 95% confidence intervals, signify the Ct products required to achieve 50% and 99% lethality of the termites, respectively. ^b^ The slope represents the correlation between the Ct products and the lethality of the termites. SE: standard error. ^c^ degree of freedom. ^d^ *X*^2^ based on pooling of data with low expectation.

**Table 2:** The Ct products of sulfuryl fluoride depending on the loading ratios of wood (10, 30, and 50%) and the moisture content at 5 °C and 23 °C.

Temp (°C)	Loading Ratio (*v*/*v* %)	Ct Products (mg h/L)		
		Dry	Wet *	*p*
23	10	665.74 ± 5.89	712.90 ± 20.95	0.214
	30	692.68 ± 40.96	743.16 ± 6.04	0.387
	50	732.25 ± 13.96	757.34 ± 26.50	0.339
	*p*	0.250	0.336	
	*F*	1.761	1.313	
5	10	900.05 ± 38.78	781.23 ± 38.76a	0.081
	30	880.84 ± 5.13	779.23 ± 28.46ab	0.094
	50	805.57 ± 42.36	985.32 ± 61.68ac	0.202
	*p*	0.187	0.028	
	*F*	2.250	6.877	

* Data are presented as the mean ± standard error. Means followed by the same letters within a column are not significantly different in the Scheffe test (*p* > 0.05) (SPSS 12.0K).

**Table 3:** Efficacy of sulfuryl fluoride fumigation on *Reticulitermes speratus* in wooden cubes at 23 °C for 24 h in laboratory trials (6.9 L).

No. of Termites	LCt_50_^a^ (95% CI, mg h/L)	LCt_99_^a^ (95% CI, mg h/L)	Slope ± SE ^b^	*df* ^c^	*X* ^2 d^
540	31.59 (30.47–32.70)	53.34 (49.22–59.63)	10.23 ± 0.91	22	127.2

^a^ The LCt_50_ and LCt_99_ values, along with the 95% confidence intervals, signify the Ct products required to achieve 50% and 99% lethality of the termites, respectively. ^b^ The slope represents the correlation between the Ct products and the lethality of the termites. SE: standard error. ^c^ degree of freedom. ^d^ *X*^2^ based on pooling of data with low expectation.

## Data Availability

All data supporting the findings of this study are available from the corresponding authors upon reasonable request.
